# Higher levels of Zidovudine resistant HIV in the colon compared to blood and other gastrointestinal compartments in HIV infection

**DOI:** 10.1186/1742-4690-7-74

**Published:** 2010-09-13

**Authors:** Guido van Marle, Deirdre L Church, Kali D Nunweiler, Kris Cannon, Mark A Wainberg, M John Gill

**Affiliations:** 1 Department of Microbiology and Infectious Diseases, University of Calgary, Calgary, Alberta, Canada; 2 Department of Pathology and Laboratory Medicine, University of Calgary, Calgary, Alberta, Canada; 3 Department of Medicine, University of Calgary, Calgary, Alberta, Canada; 4 Calgary Laboratory Services, Calgary, Alberta, Canada; 5 McGill University AIDS Centre, Lady Davis Institute-Jewish General Hospital, Montreal, Quebec, Canada; 6 Department of Microbiology and Immunology, McGill University, Montreal, Quebec, Canada

## Abstract

**Background:**

The gut-associated lymphoid tissue (GALT) is the largest lymphoid organ infected by human immunodeficiency virus type 1 (HIV-1). It serves as a viral reservoir and host-pathogen interface in infection. This study examined whether different parts of the gut and peripheral blood lymphocytes (PBL) contain different drug-resistant HIV-1 variants.

**Methods:**

Gut biopsies (esophagus, stomach, duodenum and colon) and PBL were obtained from 8 HIV-1 infected preHAART (highly active antiretroviral therapy) patients at three visits over 18 months. Patients received AZT, ddI or combinations of AZT/ddI. HIV-1 Reverse transcriptase (RT)-coding sequences were amplified from viral DNA obtained from gut tissues and PBL, using nested PCR. The PCR fragments were cloned and sequenced. The resulting sequences were subjected to phylogenetic analyses, and antiretroviral drug mutations were identified.

**Results:**

Phylogenetic and drug mutation analyses revealed differential distribution of drug resistant mutations in the gut within patients. The level of drug-resistance conferred by the RT sequences was significantly different between different gut tissues and PBL, and varied with antiretroviral therapy. The sequences conferring the highest level of drug-resistance to AZT were found in the colon.

**Conclusion:**

This study confirms that different drug-resistant HIV-1 variants are present in different gut tissues, and it is the first report to document that particular gut tissues may select for drug resistant HIV-1 variants.

## Introduction

Science has been confronted with the problem of drug-resistance virtually since the introduction of the first antiretroviral drugs to treat infection by human immunodeficiency virus type 1 (HIV-1) (reviewed in [1]). The first approach to antiretroviral therapy (ART) used single nucleoside reverse transcriptase inhibitors (NRTIs) which were found to select for drug-resistant variants very quickly [1,2]. The development of many new NRTI, non-nucleoside RT inhibitors (NNRTI), and protease inhibitors (PI) offered additional treatment options in cases of drug-resistance. It also offered the possibility of combination therapies (i.e. highly active antiretroviral therapy (HAART)) able to suppress HIV replication and reduce the likelihood of developing drug-resistance [1-3].

The ability of HIV-1 to rapidly develop drug-resistance is linked to its highly divergent nature as a result of the error-prone reverse transcription step in its life cycle [4]. Due to the high mutation rate, HIV-1 exists in the infected individual as a collection of many different viral variants, also known as a quasi-species [5]. The extent of quasi-species diversity during infection is amongst others affected by factors such as viral fitness, availability of cells for infection, selective pressure from antiretroviral therapy, duration of infection, and host immune responses [5-8].

Studies of patients on antiretroviral therapy revealed that viral sequences continued to evolve in genes not targeted by the drugs, despite successful suppressive therapy [9-11]. This phenomenon can be explained by continued viral replication in other tissues and/or cell compartments due to inefficient action or penetration of the antiretroviral drugs (ARVs) in these compartments. These inefficiently targeted compartments are referred to as sanctuary sites (reviewed in [12-14]). The central nervous system (CNS) is well known as a sanctuary site, because certain antiretroviral drugs do not easily cross the blood-brain barrier [13]. Recent studies postulated the gut may also be an important sanctuary site, where HIV-1 can persist despite successful antiviral therapy [15,16]. This is consistent with observations in the SIV model [17]. The gut-associated lymphoid tissue (GALT) is known as a major site for viral replication, CD4^+ ^T-cell depletion, and immune dysfunction [18-22]. However, relatively little is known about the distribution of HIV-1 antiretroviral drug-resistance across different parts of the gastrointestinal (GI) tract. We recently showed that HIV-1 quasi-species varied within different parts of the GI tract of pre-HAART patients, indicating that HIV-1 replication in the gut is compartmentalized [23]. Now, we have extended these observations to show that variability exists in the distribution of drug-resistant variants in different gut tissues and peripheral blood lymphocytes of these pre-HAART patients. The number of drug-resistant HIV variants differed in the colon compared to blood and other gut tissues, depending on the antiretroviral therapy received. This suggests that antiretroviral drug-resistance is highly variable in the different gut compartments.

## Results

### Diversity of the HIV-1 RT-coding region in different gut tissues

The samples were obtained from a preHAART cohort study of HIV-1 seropositive men who have sex with men (MSM) [24,25]. The 8 patients in the current report were used in an earlier study of HIV-1 diversity in the gut [23]. For the current study, gut and peripheral blood lymphocyte (PBL) samples from these 8 patients from 3 subsequent visits over 18 months were used (Table 1). All patients were on mono- or dual therapies of primarily AZT (azidothymidine, zidovudine) and ddI (dideoxyinosine). One patient (#42) died during the study and only samples from the first visit were available. This patient was still included in our analyses as a patient with end stage disease and suspected drug resistance. In addition, five patients (#1, #3, #7, #8, #19) were still alive at the time of this study (2007) and received HAART. For patients #3, #7, #8, #19, year 2007 PBL samples (indicated as Visit 2007) were collected, and drug-resistance mutations were assessed to get insight in the drug resistance mutations 15 years after the original visit. HIV-1 viral sequences were most consistently amplified from DNA from most PBL and biopsy tissues, using our nested PCR protocol. Therefore, our analyses focused on these viral DNA derived sequences. For some patient visits RT-coding sequences from only two gut-tissues and PBL could be obtained, in particular for visit 1 (Additional File 1). In total, around 1000 RT-coding sequences were obtained and analyzed.

**Table 1:** Patient Information

Patient	**Date HIV**^**+**^	**Date Death**^**1**^	**Prior Therapy**^**2**^	Visit 1				Visit 2				Visit 3			
				Date	**VL**^**3**^	**CD4 Count**^**4**^	Therapy	Date	**VL**^**3**^	**CD4 Count**^**4**^	Therapy	Date	**VL**^**3**^	**CD4 Count**^**4**^	Therapy
**#1**	Jun. 1 1986	--	AZT	Apr. 7 1993	2.7	264	ddI, AZT	Jan. 19 1994	2.4	210	ddI, AZT	Oct. 26 1994	3.5	190	AZT
**#2**	Jan. 1 1989	Oct. 16 1994	ddI	Apr. 7 1993	6.4	187	ddI	Jan. 26 1994	5.6	40	D4T	Sept. 14 1994	5.8	18	None
#3*	Nov. 1 1989	--	ddI	Apr. 6 1993	4.3	144	ddI	Jan. 26 1994	5.3	162	AZT	Sept. 13 1994	5.6	77	AZT
#7*	Jun. 1 1987	--	AZT	Apr. 21 1993	4.5	270	ddI	Jan. 26 1994	4.9	234	ddI	Mar. 9 1995	4.5	146	AZT
#8*	Oct. 1 1988	--	AZT	Apr. 21 1993	3.3	475	ddI	Jan. 12 1994	3.5	338	ddI	Nov. 16 1994	4.4	325	ddI
#19*	Jan. 1 1991	--	ddI	Jun. 2 1993	3.9	77	ddI	Jan. 26 1994	5.3	22	AZT	Nov. 14 1994	4.7	42	AZT
**#60**	Dec. 1 1988	Apr. 3 1998	AZT	Sept. 14 1994	4.5	48	AZT	Jun. 14 1995	4.9	51	AZT	Feb. 28 1996	5.3	21	ddI
**#42**	Nov. 1 1989	Oct. 21 1993	AZT, ddC	Oct. 6 1993	5.6	9	None								

^1^Patient #42 passed away during the study and only visit 1 samples were available

^2^Antiretroviral therapy in the 6 months preceding visit 11

^3^VL - log plasma viral load in log_10 _(copies)/mL

^4^CD4^+ ^cell counts in cells/μL

*Patients for which a 2007 PBL sample was analyzed

**Figure 1:**
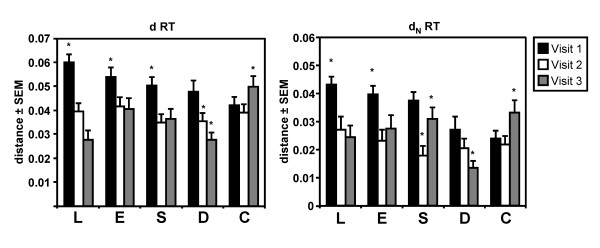
**Viral interpatient diversity of the RT-coding region in the gut tissues (esophagus (E), stomach (S), duodenum (D), colon (C)) and PBL (L) of HIV-1 infected patients at different visits**. Viral RT-coding sequences tended to a more conserved sequence among patients in the esophagus, stomach, duodenum and PBL, as reflected by the lower mean total distance (d) between patients, while the sequences in the colon became more diverse over time. Similarly, the decrease in mean total non-synonomous distance (d_N_, i.e. amino acid changing mutations) for PBL and duodenum suggested evolution towards more conserved RT protein sequences over time among patients, while the increased d_N _reflected the RT protein sequence becoming more diverse over time in the colon among patients. These observations indicated that the RT-coding region evolved differently in the different gut tissues and PBL in this group of patients. (* *p *< 0.05, Dunnett C post-hoc analysis)

The mean total (d), and nonsynonymous (d_N_) pair-wise distances between patients were calculated for the RT-coding sequences obtained from PBL, esophagus, stomach, duodenum, and colon for all patients at all visits (Figure 1). Although we were not able to obtain sequences from the duodenum and colon for visit 1 for a number of patients, the overall interpatient distances (d) of RT coding sequences tended to decrease (*p *< 0.05) at the last visit for sequences derived from the PBL, esophagus, stomach, and the duodenum (Figure 1). This suggested evolution towards a more conserved RT coding region between patients in these tissues in this particular sample of patients. In contrast, the overall interpatient distance of the RT-coding sequences in the colon increased over time (*p *< 0.05) (Figure 1). A decrease in the d_N _values (i.e. codon/amino acid changing substitutions) (*p *< 0.05) towards the last visit between the patients was observed for the RT-coding region for both PBL and duodenum, while in the esophagus and stomach the d_N _decreased but fluctuated over time. In the colon the d_N _values increased (Figure 1), suggesting greater interpatient diversity in RT coding sequences in the colon in this group of patients.

### Compartmentalization of antiretroviral drug-resistance in the gut

**Figure 2:**
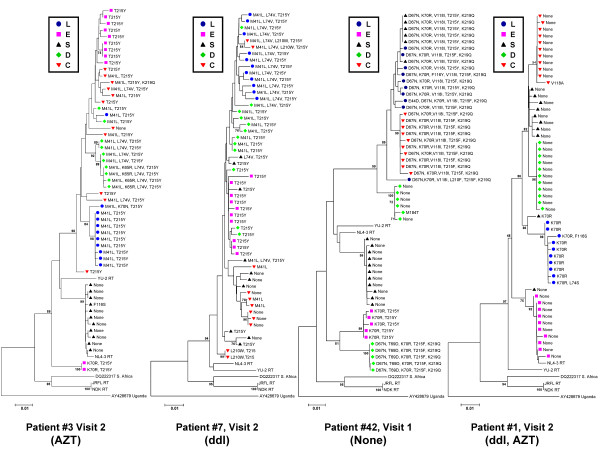
**Representative bootstrap Neighbor-Joining trees of RT-coding sequences obtained from gut tissues (esophagus (E), stomach (S), duodenum (D), colon (C)) and PBL (L) (indicated by different shapes and shading)**. RT sequences grouped by individual gut tissue and PBL to varying degrees in the different patients. Upon closer examination of the drug-resistance mutations indicated at each branch, grouping of resistance mutations by gut tissue and PBL was observed. Differences in drug-resistance mutations were found in the different tissues and PBL. Similar differences were observed for RT sequences recovered from the tissues of other patients, indicating difference in distribution of drug-resistance in the gut. (Bootstrap values > 70 are indicated.)

The RT sequences were subsequently subjected to phylogenetic analyses. Neighbour-joining trees of all RT sequences and bootstrap analysis revealed clustering of some sequences by tissue and patient (bootstrap values > 70). As we previously reported such clustering was not consistent for most of the sequences [23](data not shown). Bootstrap analyses of RT sequences by individual patient and visit, revealed more consistent clustering on the basis of tissue (bootstrap values > 70), although this also varied by patient (Figure 2). The representative neighbour-joining trees for the RT sequences for patient #3 (Visit 2), #42 (Visit 1), and #1 (Visit 2) revealed clustering of sequences (bootstrap values > 70) on the basis of tissue (Figure 2). Similar trees were obtained for the other patients and visits (data not shown). Further analysis of the clustering pattern using the Slatkin-Maddison test [26-28] revealed that there was no consistent significant compartmentalization of the RT sequences. However, many sequences grouped together by tissue in the phylogenetic trees (for example patient #7, Visit 2, Figure 2).

**Figure 3:**
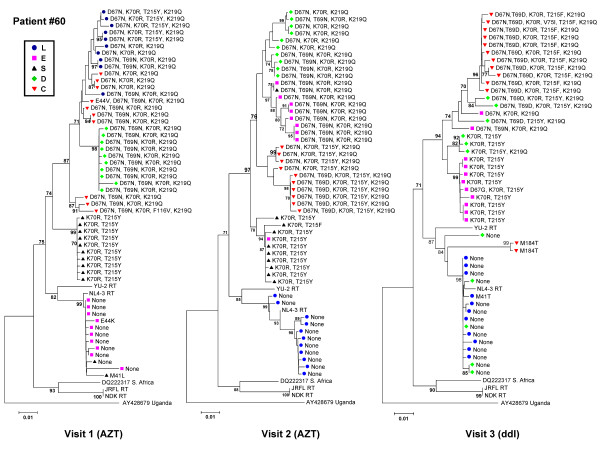
**Bootstrap Neighbor-Joining trees of the RT-coding sequences of patient #60 at visits 1, 2 and 3**. Differences in drug-resistance mutations (indicated at the tree branches) and grouping of RT-coding sequences was observed. However, at all visits differences were observed in the drug-resistance mutations between the various tissues, consistent with differential distribution of drug-resistance in the gut. Similar results were obtained for the RT-coding sequence of the other patients. (Bootstrap values > 70 are indicated.)

We analyzed the presence of mutations associated with NRTI resistance using the Stanford HIV-1 Drug-Resistance Database [29]. As shown in Figure 2, distinct drug-resistance mutations were found in each tissue compartment for patient #3 (Visit 2), consistent with grouping together of the nucleic acid sequences. For patient #1 (visit 2), we observed grouping of sequences by tissue, but very few drug-resistance mutations, probably due to the greater efficiency of therapy with two NRTIs (AZT and ddI). For patient #42 (Visit 1), various drug resistance mutations were found in the stomach, colon, and PBL, which was consistent with the suspected viral failure due to antiviral drug resistance. Different or no mutations were present in the duodenum, stomach and esophagus of this patient suggesting that antiretroviral drug resistance can differ significantly among tissues. Although the clustering of RT sequences of Patient #7 (Visit 2) was not indicative of compartmentalization according to the Slatkin-Maddison criteria, the different tissue compartments could still be separated and grouped based on drug-resistance mutations. This observation was consistent for all patients and visits, as illustrated for patient #60 in Figure 3. Repeating the phylogenetic analyses after removing the drug resistance conferring sites from the sequences resulted in the same tree topologies (data not shown), indicating that the drug resistance conferring sites were not solely responsible for the observed clustering of RT sequences by tissue.

These observations strongly suggest a differential distribution of antiretroviral drug-resistance in the different gut tissues, with drug-resistance mutations differing from those observed in the blood. These observations were further corroborated by sorting drug-mutations by tissue compartment (summarized in Table 2 and Additional File 1), indicating drug-resistance mutations differed significantly between tissues within each patient (*p *< 0.05 Chi-square test), and varied over time (*p *< 0.05, Chi-square test). Furthermore, the different tissues also differed significantly in distribution of drug-resistance mutations in the viral quasi-species (*p *< 0.05 Chi-square test). Our analysis revealed no evidence for a preferential presence of any specific drug-resistance mutations for any individual tissue compartment.

**Table 2:** Drug resistance mutations by tissue source

Patient #7	PBL	Esophagus	Stomach	Duodenum	Colon
**Visit 1**					

	None (100%)	None (20%)	None (25%	ND	ND
**ddI**		***T215Y*** (20%)	L74V (8.3%)		
		***M41L, T215Y*** (40%)	***T215Y ***(16.7%)		
		***M41L***, T69N, ***T215Y ***(20%)	L74V, ***T215Y*** (50%)		

**Visit 2**					

	***M41L***, L74V, ***T215Y ***(100%)	***T215Y ***(100%)	None (45.5%)	***M41L***, ***T215Y***(54.5%)	***M41L***(33.3%)
**ddI**			***M41L***, L74V, ***T215Y ***(9.1%)	***T215Y ***(27.3%)	L210W, ***T215Y***(22.2%)
			***T215Y ***(36.4%)	***M41L***, L74V, ***T215Y ***(18.2%)	None (44.4%)
			L74V, ***T215Y ***(9.1%)		

**Visit 3**					

	None (87.5%)	***M41L ***(42.9%)	***T215Y***(93.8%)	***M41L ***(18.2%)	***M41L***, L210W, ***T215Y***(71.4%)
**AZT**	F77S (12.5%)	***T215Y ***(57.1%)	L210F, ***T215Y***(6.25%)	None (81.8%)	***M41L ***(14.3%)
					***M41L***, V75G (14.3%)

**Visit 2007**					

	None (100%)				
**HAART**					
					

**Patient #60**	**PBL**	**Esophagus**	**Stomach**	**Duodenum**	**Colon**

**Visit 1**					

	D67N, K70R, ***T215Y***, K219Q (80%)	None (90%)	None (8.3%)	D67N, T69N, K70R, K219Q (100%)	D67N, K70R, K219Q (30%)
**AZT**	D67N, K70R, K219Q (20%)	E44K (10%)	***M41L ***(8.3%)		D67N, T69N, K70R, K219Q (50%)
			K70R, ***T215Y***(83.3%)		D67N, T69N, K70R, F116V, K219Q (10%)
					E44V, D67N, T69N, K70R, K219Q (10%)

**Visit 2**					

	None 100%	D67N, T69N, K70R, K219Q (90%)	K70R, ***T215F ***(10%)	D67N, K70R, K219Q (50%)	D67N, K70R, ***T215Y***, K219Q (40%)
**AZT**		K70R, ***T215Y*** (10%)	D67N, T69N, K70R, K219Q (10%)	D67N, T69N, K70R, K219Q (50%)	D67N, T69D, K70R, ***T215Y***, K219Q (60%)
			K70R, ***T215Y ***(80%)		

**Visit 3**					

	None (90.9%)	D67N, T69N, K70R, K219Q (10%)	ND	None (45.5%)	M184T (15.4%)
**ddI**	M41T (9.1%)	D67G, K70R, ***T215Y*** (10%)		K70R, ***T215Y***(18.2%)	D67N, T69D, K70R, ***T215F***, K219Q (76.9%)
		D67N, K70R, K219Q (10%)		K70R, ***T215Y***, K219Q (9.1%)	D67N, T69D, K70R, V75I, ***T215F***, K219Q (7.7%)
		K70R, ***T215Y ***(70%)		D67N, K70R, ***T215Y***, K219Q (9.1%)	
				D67N, T69D, K70R, ***T215Y***, K219Q (9.1%)	
				D67N, T69N, K70R, ***T215Y***, K219Q (9.1%)	

**Patient #19**	**PBL**	**Esophagus**	**Stomach**	**Duodenum**	**Colon**

**Visit 1**					

	M41I, E44K, D67N, L74V (100%)	K70R (10%)	M41I, E44K, D67N, L74V (40%)	ND	ND
**AZT**		E44G (10%)	None (60%)		
		F77S (10%)	None (60%)		
		D67N, K70R, V118I, ***T215Y***, K219Q (10%)			

**Visit 2**					

	***M41L***, ***T215Y ***(100%)	None (77.8%)	***T215Y ***(100%)	None (50%)	F116K (14.3%)
**AZT**		D67G (11.1%)		L74V (50%)	None (85.7%)
		T215A (11.1%)			

**Visit 3**					

	***T215Y***(20%)	***M41L***, ***T215Y ***(100%)	None (25%)	None (77.8%)	***M41L***, L74V, ***T215Y ***(66.7%)
**AZT**	***M41L***, ***T215Y*** (80%)		K70R (50%)	***T215Y ***(11.1%)	None (33.3%)
			L210W, ***T215Y ***(16.7%)	***M41L***, D67G (11.1%)	
			***M41L***, L210W, ***T215Y*** (8.3%)		

**Visit 2007**					

	None (81.8%)				
**HAART**	***M41L***, E44D, T215C (18.2%)				
					
**Patient #42**	**PBL**	**Esophagus**	**Stomach**	**Duodenum**	**Colon**

**Visit 1**					

	D67N, K70R, V118I, ***T215Y***, K219Q (70%)	K70R, ***T215Y***(100%)	None (58.3%)	M184T (9.1%)	D67N, K70R, V118I, ***T215F***, K219Q (100%)
**none**	D67N, K70R, V118I, L210F, ***T215F***, K219Q (10%)		D67N, K70R, V118I, ***T215Y***, K219Q (41.7%)	D67N, T69D, K70R, ***T215F***, K219Q (45.5.%)	
	D67N, K70R, F116Y, V118I, ***T215F***, K219Q (10%)			None (45.5%)	
	E44D, D67N, K70R, V118I, ***T215F***, K219Q (10%)				

*ND - no viral sequences detected. Primary drug resistance mutations associated with high levels of drug resistance indicated in italics

Table 2 also shows the drug-resistance mutation profile of the PBL samples for two surviving patients currently on HAART, collected in 2007, 15 years after the original study. Again, drug-resistance mutations differed from the original historical samples (*p *< 0.05 Chi-square test), and similar results were obtained for the other two surviving patients (Additional File 1). This analysis confirms that the changes observed in the viral DNA samples were the result of changes in the viral population close to the time of sampling and not the result of picking up viral DNA sequences that were archived over many years.

### Different drug-resistance levels in different parts of the gut

**Figure 4:**
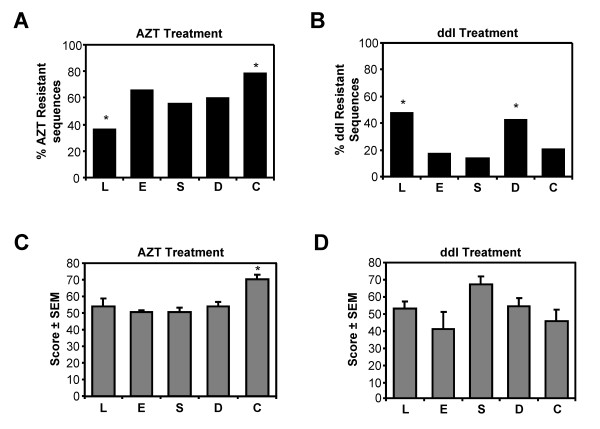
**Analysis of the effects of AZT and ddI treatment on drug-resistance in esophagus (E), stomach (S), duodenum (D), colon (C) and PBL (L)**. Resistance mutations were recorded and scored using the Stanford Drug-Resistance Database for level of drug-resistance. Sequences with intermediate to high-level resistance for AZT, or ddI were considered drug-resistant. The number of drug-resistance sequences recovered after AZT **(A) **or ddI **(B) **treatment in each tissue was expressed as the percentage of all sequences recovered from the tissue. Different numbers of AZT and ddI resistant sequences were found in the gut tissues and PBL following AZT treatment and ddI treatment, respectively (* *p *< 0.05, Pearson chi-square test) The average drug-resistance score for each drug also varied in each tissue. AZT drug resistance scores were the highest in the colon following AZT treatment **(C)**. However, ddI resistance scores did not differ significantly in the different tissues following ddI treatment **(D)**. These observations are consistent with differential distribution of antiretroviral drug-resistance in the gut, and indicated that the AZT and ddI treatment affected each tissue differently (* *p *< 0.05, Tukeys HSD post-hoc analysis).

The development of drug-resistance is dependent on the drugs used in therapy. We analyzed the percentage of drug-resistant sequences and the average drug-resistance score for all sequences recovered from each tissue, taking into account the antiretroviral therapy received prior to the time samples were collected (Figure 4). The Stanford database was used to determine the drug-resistance score for each RT sequence. RT sequences with a drug-resistance score ≥ 30 were designated drug-resistant. Following AZT or ddI treatment, different numbers of respectively AZT- or ddI-resistant RT-coding sequences were found in the GI tissues (esophagus, stomach, duodenum and colon) and PBL (i.e. blood) (*p *< 0.05, Figure 4A and 4B). Thus, the distribution of drug-resistant sequences is diverse, and antiretroviral therapy selects for different numbers of drug-resistant HIV-1 variants in each tissue.

Next, we analyzed the average drug-resistance score of all drug-resistant RT sequences (i.e drug-resistance score ≥ 30) among the different tissues following AZT or ddI treatment (Figure 4C and 4D). This analysis revealed that RT sequences with the highest drug-resistance score for AZT were recovered from the colon (Figure 4C, *p *< 0.05). No significant differences in ddI resistance scores were observed following ddI treatment, although they tended to be higher in the stomach (Figure 4D). Together with our other observations, these results suggested that antiretroviral therapies (AZT and ddI) affected each gut tissue compartment differently, and that AZT preferentially selected for more AZT resistant HIV-1 variants in the colon.

## Discussion

The presence of HIV-1 antiretroviral drug-resistance in different tissues, such as the CNS, has been well document [30-33]. However, little is known about HIV-1 antiretroviral drug-resistance in different tissues of the gut, despite its importance as a reservoir for viral replication and a host pathogen interphase in HIV/AIDS [18-22]. To provide insight into the potential distribution of HIV-1 drug-resistance at different locations in the gut (esophagus, stomach, duodenum and colorectum) and in peripheral blood lymphocytes (PBL), we analyzed the RT sequences from 8 HIV-1 infected patients. Our previous study on compartmentalization of HIV-1 replication revealed a greater compartmentalization of the viral quasi-species for the Nef region compared to the RT-coding region [23]. Similarly, the current study indicated that compartmentalization is less prominent for the RT coding region. The bootstrap analyses clearly indicated clustering of RT sequences by tissues in a number of patients but not all. Moreover, the clustering could not be considered a sign of significant compartmentalization of RT sequences in the GI tract according to the criteria of the Slatkin-Maddison test. However, the current study clearly showed that patterns of HIV-1 drug-resistance significantly vary across different gut compartments, distinct from what is found in blood (i.e. PBL). This is indicative of a differential distribution of HIV-1 antiretroviral drug-resistance in the GI-tract.

Varying viral diversity was observed for the RT-coding region in gut and PBL over time. Despite the fact that we were unable to obtain sequences for all lower GI tissues for a number of patients at visit 1, we observed a tendency towards a more conserved RT-coding region in the PBL, esophagus, stomach and duodenum between patients at the later visits. This may be a sign of adaptation of the virus to the different tissues, as there are some indications the RT-protein might affect cell tropism [34,35]. In addition, the host immune response could shape viral evolution and select for particular viral sequences in different tissues [36-40]. In contrast, viral diversity for the RT-coding region between patients increased over time in the colon. Although, we did not study variation over time and only assessed one isolated visit in our previous study on the compartmentalization of the gut viral reservoir [23], the data of that study also suggested an increased diversity in both the Nef- and RT-coding regions in the colon. We explained this increased viral diversity by the higher levels of HIV-1 replication that we and others have observed in the colon [9-11,23]. Probably in part due to the activated state of the GI tract in HIV-1 infection, lymphoid cells obtained from the GI-tract are very susceptible to HIV infection compared to blood or other tissue lymphocytes allowing for an increased viral replication [41-43]. The increased error prone replication would result in higher viral diversity. Although these findings corroborate our previous observations, we did observe that viral diversity between patients fluctuated to various degrees over time among the different tissues, indicating HIV-1 quasi-species evolution in the different compartments is dynamic.

For our current study, we were only able to consistently amplify HIV RT sequences from the integrated and nonintegrated viral DNA found in total tissue DNA. Therefore, our study was restricted to an analysis of HIV drug resistance of the banked viral reservoir and potentially not actively replicating viruses. Despite this limitation our data clearly indicated that antiviral drug resistant mutations are easily detected in the gut viral DNA reservoir. Furthermore, our data revealed that the viral gut reservoir is variable and dynamic. Significant changes together with selection for antiviral drug resistance occurred within a matter of weeks or months under continuous antiviral therapy. These banked viral reservoirs are clinically significant as they could be an important source of drug resistant viruses.

As in our previous study [23], analyses of all RT-coding sequences did not reveal the same pattern of clustering by tissue that we observed for the Nef encoding region. The bootstrap analysis of sequences by individual patient and visit revealed varying degrees of clustering of sequences by tissue among the different patients, but this clustering did not pass the Slatkin-Maddison test for compartmentalization. The latter would suggest that the different gut tissues are not strictly isolated reservoirs and viruses are exchanged between the different compartments, similar to what has been reported for HIV-1 in blood and lung compartments in *Mycobacterium tuberculosis *co-infected individuals [44]. However, NRTI drug-resistance mutations grouped by tissue compartment, which is consistent with compartmentalization of HIV replication in the gut [23]. Moreover, drug-resistance mutations varied among various tissues, and differed from those in the blood (i.e. PBL). The levels of drug-resistance also varied across the different tissues, as indicated by the number of drug-resistant RT sequences recovered from the gut tissues and PBL, and the average resistance scores for AZT and ddI. The drugs could target the tissues with different efficiency, thereby selecting differentially for drug-resistant viruses in each tissue. Alternatively, the immune activated state of the GI tract during HIV-1 infection could also alter drug metabolism and turnover in the different gut tissues. Other studies have observed differential distribution of HIV sequences and antiviral drug resistance amongst different immune cells in the blood depending on the patient [45,46]. It is therefore possible that the differential distribution of different populations of immune cells in the gut is underlying the differential distribution of drug resistance in our study. For the immune cells in the blood compartment, Potter *et al. *[45] postulated that differences in drug penetration in the different cells and different cell turn-over due to, for instance, differences in viremea or inflammatory response could alter cell distribution. This could also play a role in each gut tissue, and alter viral populations and drug resistance in a patient and tissue dependent fashion.

Based on our observations, one may conclude that AZT resistant viruses may arise first in the colon, and then start seeding the PBL and other gut tissues. The current data does not allow us to determine this unequivocally and further studies will be necessary. Our data did suggest that the colon selected for highly drug-resistant viruses. This could be due to the different antiretroviral drug-concentrations in the different tissues. Studies in rats have shown that after oral administration the intestinal absorption of zidovudine is lower in the colon compared to other parts of the intestinal tract (i.e. duodenum and jejunum)[47]. To our knowledge it is unknown how this effects drug concentrations in the colon, although in prenatal foetal rats higher zidovudine concentrations have been reported in the colon compared to plasma [48]. The higher number of target immune cells in the colon compared to the esophagus, stomach and duodenum [49-52], together with these altered drug concentrations could facilitate the evolution of highly drug-resistant viruses. Similarly, as part of the adaptation processes of HIV-1 to these tissues, certain mutations in the RT protein may be required that also happen to affect antiretroviral drug-resistance. The increased level of AZT resistance in the colon is of interest as various studies have shown that viral RNA/loads can remain higher in the colon under antiretroviral viral therapy, even when the plasma viral loads are effectively reduced [9,10,53-56]. Again further studies will be necessary, but our observations would explain why this is the case.

Our analysis focused on the primary drug resistance mutations in the main body of the RT encoding region. The sequencing method used did not analyze either the connection or RNase H domains of RT, both of which are known to contain sites that can affect levels of resistance to AZT [57-59]. It would be of interest to examine how this important part of the RT region evolves in the different gut tissues, as our current analyses may actually underestimate AZT resistance in the GI tract.

Finally, the patient samples for this study were collected during the preHAART era (1993-1996). Our analysis of antiretroviral drug-resistance in different parts of the gut in this period of the HIV epidemic is extremely relevant in the current era of HAART. Suboptimal treatment conditions still exist, in part due to patient noncompliance and toxicity of antiretroviral drugs. The data gathered from our studies about preHAART HIV-1 infection of the gut is also of importance for the HIV-1 epidemic in the developing world, where comprehensive HAART regimens may not be consistently available, and the proposed antiviral strategies may not be fully suppressive. Moreover, our observations are also relevant for other HIV-1 subtypes as they also have been shown to replicate differentially in the GALT (reviewed in [60]). The importance of viral reservoirs or archives in antiretroviral therapy is illustrated by recent observations in the context of antiretroviral therapy to reduce mother-to-child transmission. A single dose treatment with the NNRTI inhibitor nevirapine was already enough to establish nevirapine resistance in the latent cell reservoirs in the blood of the HIV infected mother [61]. This could complicate subsequent ART or HAART treatments due to preexisting drug resistance. A better understanding of the evolution of antiretroviral drug-resistance in the different gut tissues and other cell compartments will help optimize antiretroviral therapies in both developed and developing countries.

## Conclusions

It has been proposed that HIV-1 can "hide" from antiretroviral therapy in the gut, and drug-resistance may be compartmentalized [15,16,62]. Our results showed that antiretroviral resistance differed among the different gut tissues and is highly variable. More importantly it showed that drug-resistance in the gut can be completely different from what is observed in the periphery (i.e. blood). The differential distribution of antiretroviral drug-resistance in the gut and the differential selection for drug-resistant viruses in the gut; support the hypothesis that the gut could act as "hide-out" from antiretroviral therapy [15,16].

## Methods

### Patients and gut biopsy samples

Samples for this study had been collected from patients enrolled in a previous cohort study of HIV-1 seropositive men who have sex with men (MSM) followed at the Southern Alberta Clinic (SAC), Calgary, Alberta from 1993-1996 [24,25]. All protocols were reviewed and approved by the Office of Medical Bioethics of the University of Calgary and patients signed informed consent documentation upon enrolment [25]. Patients were prospectively followed and laboratory analyses included plasma viral load and CD4^+ ^cell counts at each study visit. At approximate 6 month intervals, upper and lower gastrointestinal endoscopies were performed and biopsies were collected from the esophagus, stomach, duodenum, and colon and stored at -70°C [24]. Peripheral blood lymphocytes (PBL) were isolated from blood and stored in liquid nitrogen [24,25]. This cohort was recruited prior to the introduction of HAART at the SAC in 1997. The 8 patients described in this study were used earlier in a study on HIV-1 diversity in the gut (Table 1.) [23]. Gut tissue and PBL samples from three consecutive visits were analyzed, covering a time period of 18 months. Disease progressed in all patients (mean age 36 yrs, range 30-44 yrs), with an average CD4^+ ^cell count of 125 ± 122 cells/μl, and plasma viral load of 4.0 ± 0.8 log_10 _copies/ml at the last visit (Table 1). During the time interval studied, the patients received monotherapy or dual therapy with the NRTIs: azidothymidine (AZT, zidovudine), dideoxyinosine (ddI) prior to and during the study period (Table 1). One patient (#42) also received dideoxycitidine (ddC) in combination with AZT during the period preceding the study and died after the first visit (Table 1). Five patients were still alive at the time of this study (2007) and received HAART. PBLs from four of these patients (#3, #7, #8, #19) were collected, and drug-resistance mutations (15 years after the original study) were assessed, and are indicated as Visit 2007.

### DNA Isolation and PCR Amplification of Viral Sequences from Gut Biopsies

Total DNA was isolated from tissue using Trizol Reagent (Invitrogen, Burlington, ON), and HIV-1 reverse transcriptase (RT)-coding sequences (at nt. 2604-3251) were amplified from viral DNA by nested PCR as described previously [23]. The amplification of integrated and nonintegrated viral DNA ensured that expressed, dormant and/or 'banked' varieties were included in our analysis [63-66]. Briefly, the nested PCR protocol consisted of denaturation at 94°C for 5 min, 45 cycles of 1 min at 95°C, 1 min at the annealing temperature of the primer set used, 2 min at 70°C, and a final extension step of 10 min at 70°C. The primers used for the first round were RT 2470 5'-GTA CAG TAT TAG GAC CTA CAC CTG-3' and RT 3261 5'-ATC AGG ATG GAG TTC ATA ACC CAT CCA-3' (T_m _= 55°C), and for the second round consisted of RT 2604 5'-CCA AAA GTT AAA CAA TGG CCA TTG ACA-3' and RT 3251 5'-AGT TCA TAA CCC ATC CAA AG-3' (T_m _= 55°C). Both primary and nested PCR reactions were performed with a high fidelity *Taq *polymerase to reduce the incorporation of mutations during amplification. To avoid selective amplification of the most dominant viral sequences at the expense of less frequent viral sequences due to high template concentrations or amplification of single viral DNA copies due to low template concentrations, optimal template concentrations were determined by dilution experiments. We used 2 to 10 fold dilutions of template DNA (initial input 0.2 μg), and the dilutions that yielded the most abundant PCR products were used for analyses. To prevent contamination with amplicons, DNA isolation, PCR amplifications and subsequent cloning steps, were performed in separated rooms and laboratory areas. Negative (no viral DNA) and positive (plasmid containing proviral DNA of HIV-1 strain NL4-3) controls were included during all amplifications. The PCR fragments were separated and isolated from agarose gels, and directly sequenced to ensure genuine HIV-1 viral DNA had been amplified.

### Sequence Analysis

To analyze the HIV-1 quasi-species within each tissue sample, the nested PCR products identified as amplicons of genuine HIV-1 DNA were cloned into pCR2.1 TOPO linearized vector using the TA cloning kit (Invitrogen, Burlington, ON). Plasmids containing relevant inserts were purified from bacteria using a Plasmid Mini-Prep Kit (Qiagen, Mississauga, ON). The inserts were sequenced on an automated ABI sequencer (Applied Biosystems, Streetsville, ON) and a Li-Cor 4300 DNA Analysis sequencing system (Li-Cor Biosciences, Lincoln, NE) according to manufacturers' protocols. For each compartment, 5-10 clones containing HIV-1 RT fragments were sequenced. For a number of samples we repeated both PCR and subsequent sequence analysis, and similar results were obtained, indicating our approach was reproducible. Sequences have been submitted to Genbank (EF656787 to EF656965, and EU931894 to EU932684).

### Phylogenetic Analysis

The inferred amino acid sequence for the cloned DNA fragments was obtained for each sample, and screened for the integrity of coding sequences, i.e. stop codons, deletions, and/or insertions. DNA sequences were subjected to phylogenetic analysis using the Molecular Evolutionary Genetics Analysis (MEGA) version 4.0 software (http://www.megasoftware.net) [67]. Neighbour-joining trees were constructed using the Kimura-2-parameter model with 5000 replicates for the bootstrap analysis. Bootstrap values >70 were considered significant. Compartmentalization of sequences was assessed using the Slatkin-Maddison test using the Mesquite Software Package (http://mesquiteproject.org)[26-28]. Sequences of prototypic HIV-1 isolates (NL4-3, YU-2, NDK) were included to rule out contamination with laboratory strains and to root the bootstrap trees. The MEGA software was used to calculate mean total interpatient (d), non-synonymous (d_N_, codon changing substitutions), and synonymous (d_S_, non-codon changing substitutions) distances [5] for the nucleic acid sequences for each patient in regard to tissue compartments, as well as differences between patients at each visit. All distances and phylogenetic analyses were calculated after pair-wise deletion and stripping of gaps in the aligned sequences. Analysis of variance (ANOVA) and Dunnett C post-hoc tests were performed to compare the distances.

### Antiretroviral Drug-Resistance Analysis

All RT sequences were screened for drug-resistance mutations (NRTI as well as NNRTI) using the Stanford HIV-1 Drug-Resistance Database algorithm (http://hivdb.stanford.edu/) [29]. Mutations not identified to be associated with resistance were also recorded. The Stanford database assigns a numerical score to each sequence with regard to individual antiretroviral drugs, i.e. susceptible (0-9), potential-low level (10-14), low-level (15-29), intermediate (30-59), or high-level (≥60) resistance. Drug-resistance scores assigned by the database for AZT, and ddI, (as the most commonly used antiretroviral drugs in this study) were tabulated and grouped for all patients and visits. Visits of patients, in which patients did not receive therapy, or other drugs then AZT or ddI, were excluded from the analysis. For the purpose of this study, we arbitrarily assigned clones to one of two nominal categories (resistant or not resistant) based on the drug-resistance score assigned by the database. Clones scoring intermediate or high-level (≥30) were assigned to the resistant category and isolates scoring susceptible, potential-low level, or low-level (< 30) were assigned to the not resistant category. The Pearson chi-square test was used to test for differences in the number and distribution of drug-resistant mutations and sequences between tissues. ANOVA with Tukey's HSD post-hoc analysis was used to compare mean drug-resistance scores between tissue compartments.

### Statistical Analyses

All statistical analyses were performed using SPSS version 11.5 (SSPS Inc., Chicago IL), *p *values < 0.05 were considered significant.

## Competing interests

The authors declare that they have no competing interests.

## Authors' contributions

GvM, DLC, MAW MJG, and collected and analyzed data, designed the study, recruited patients and were involved in writing the paper. KN and KC were involved in designing the methods for collecting, analyzing and interpreting data, and helped putting the data and parts of the manuscript together for publication.

## Supplementary Material

Additional file 1 **Additional Data Table 1: Drug resistance mutations by tissue source**. Drug resistance mutations by tissue source for all patients in the current study. Click here for file
