# Supplementary material for: Higher levels of Zidovudine resistant HIV in the colon compared to blood and other gastrointestinal compartments in HIV infection
Source: Retrovirology. 2010 Sep 13;7:74. doi: 10.1186/1742-4690-7-74 (PMC2949729; doi:10.1186/1742-4690-7-74)
Supplement: Additional file 1 — Additional Data Table 1: Drug resistance mutations by tissue source. Drug resistance mutations by tissue source for all patients in the current study. [file 1742-4690-7-74-S1.DOC]

| **Patient #1** | **PBL** | **Esophagus** | **Stomach** | **Duodenum** | **Colon** |
| --- | --- | --- | --- | --- | --- |
| **Visit 1** |  |  |  |  |  |
|  | **K70R (81.2%)** | **None (50%)** | **None (50%)** | **None (100%)** | **ND** |
| **ddI, AZT** | **K70R, F116V, T215S, K219N (9.1%)** | ***M41L*, L74V, *T215Y* (50%)** | ***M41L*, L74V (25%)** |  |  |
|  | **K70R, M184V (9.1%)** |  | ***M41L*, L74V, *T215Y* (25%)** |  |  |
|  |  |  |  |  |  |
| **Visit 2** |  |  |  |  |  |
|  | **K70R (80%)** | **None (100%)** | **None (90%)** | **None (100%)** | **None (90.9%)** |
| **ddI, AZT** | **K70R, L74S (10%)** |  | **K70R (10%)** |  | **V118A (9.1%)** |
|  | **K70R, F116S (10%)** |  |  |  |  |
|  |  |  |  |  |  |
| **Visit 3** |  |  |  |  |  |
|  | **ND** | **ND** | ***T215Y* (100%)** | **None (100%)** | ***M41L*, *T215Y* (50%)** |
| **AZT** |  |  |  |  | ***M41L*, L74V, *T215Y* (50%)** |
|  |  |  |  |  |  |
| **Patient #2** | **PBL** | **Esophagus** | **Stomach** | **Duodenum** | **Colon** |
| **Visit 1** |  |  |  |  |  |
|  | ***T215Y* (16.7%)** | **K70R, *T215Y* (10%)** | ***T215Y* (88.9%)** | **ND** | **None (100 %)** |
| **ddI** | **K70R (83.3%)** | **L74V, *T215Y* (10%)** | **D67K, T69I, *T215Y* (11.1%)** |  |  |
|  |  | ***M41L*, L74V, *T215Y* (80%)** |  |  |  |
|  |  |  |  |  |  |
| **Visit 2** |  |  |  |  |  |
|  | **None (100%)** | **L74V (12.5%)** | **ND** | **None (100%)** | **None (54.5%)** |
| **d4T** |  | ***M41L*, L74V, *T215Y* (50%)** |  |  | ***M41L* (9.1%)** |
|  |  | ***M41L*, L74V, L210W, *T215Y* (37.5)** |  |  | **K219E (9.1%)** |
|  |  |  |  |  | ***T215Y*  (18.2%)** |
|  |  |  |  |  | ***M41L*, *T215Y* (9.1%)** |
|  |  |  |  |  |  |
| **Visit 3** |  |  |  |  |  |
|  | ***M41L*, L74V, *T215Y* (80%)** | **ND** | ***M41L*, L210W, *T215Y* (100%)** | **ND** | ***M41L*, *T215Y* (100%)** |
| **None** | ***M41L*, L74V, L210W, *T215Y* (20%)** |  |  |  |  |
|  |  |  |  |  |  |
| **Patient #3** | **PBL** | **Esophagus** | **Stomach** | **Duodenum** | **Colon** |
| **Visit 1** |  |  |  |  |  |
|  | ***T215Y* (66.8%)** | ***T215Y* (100%)** | **ND** | ***M41L*, *T215Y* (100%)** | **ND** |
| **ddI** | ***T215Y*, K219Q (8.3%)** |  |  |  |  |
|  | **F77C, *T215Y*  (8.3%)** |  |  |  |  |
|  | **K65E, *T215Y*  (8.3%)** |  |  |  |  |
|  | **F77C, *T215Y*, K219Q (8.3%)** |  |  |  |  |
|  |  |  |  |  |  |
| **Visit 2** |  |  |  |  |  |
|  | ***M41L*, *T215Y* (90.9%)** | ***T215Y* (81.8%)** | **None (90.9%)** | ***M41L*, T215Y (20%)** | **None (9.1%)** |
| **AZT** | ***M41L*, K70N, *T215Y* (9.1%)** | **K70R, *T215Y* (18.2%)** | **F116S (9.1%)** | ***M41L*, K65R, L74V, *T215Y* (40%)** | ***T215Y* (36.3%)** |
|  |  |  |  | ***M41L*, L74V, *T215Y* (40%)** | ***M41L*, *T215Y* (27.3%)** |
|  |  |  |  |  |  |
|  |  |  |  |  |  |
| **Visit 3** |  |  |  |  |  |
|  | **F77L (9.1%)** | **None (16.7%)** | **V118A (50%)** | **ND** | ***M41L* (9.1%)** |
| **AZT** | **None (90.9%)** | ***M41L*, *T215Y* (83.3%)** | **None (50%)** |  | ***M41L*, *T215Y* (54.6%)** |
|  |  |  |  |  | ***M41L*, L210W, *T215Y* (36.3%)** |
|  |  |  |  |  |  |
|  |  |  |  |  |  |
| **Visit 2007** |  |  |  |  |  |
|  | **E44K, V75I, L210W, T215N (85.7%)** |  |  |  |  |
| **HAART** | **V75I, L210W, T215N (14.3)** |  |  |  |  |
|  |  |  |  |  |  |
| **Patient #7** | **PBL** | **Esophagus** | **Stomach** | **Duodenum** | **Colon** |
| **Visit 1** |  |  |  |  |  |
|  | **None (100%)** | **None (20%)** | **None (25%)** | **ND** | **ND** |
| **ddI** |  | ***T215Y* (20%)** | **L74V (8.3%)** |  |  |
|  |  | ***M41L*, *T215Y* (40%)** | ***T215Y* (16.7%)** |  |  |
|  |  | ***M41L*, T69N, *T215Y* (20%)** | **L74V, *T215Y* (50%)** |  |  |
|  |  |  |  |  |  |
| **Visit 2** |  |  |  |  |  |
|  | ***M41L*, L74V, *T215Y* (100%)** | ***T215Y* (100%)** | **None (45.5%)** | ***M41L*, *T215Y* (54.5%)** | ***M41L* (33.3%)** |
| **ddI** |  |  | ***M41L*, L74V, *T215Y* (9.1%)** | ***T215Y* (27.3%)** | **L210W, *T215Y* (22.2%)** |
|  |  |  | ***T215Y* (36.4%)** | ***M41L*, L74V, *T215Y* (18.2%)** | **None (44.4%)** |
|  |  |  | **L74V, *T215Y* (9.1%)** |  |  |
|  |  |  |  |  |  |
| **Visit 3** |  |  |  |  |  |
|  | **None (87.5%)** | ***M41L* (42.9%)** | ***T215Y* (93.8%)** | ***M41L* (18.2%)** | ***M41L*, L210W, *T215Y (*71.4%)** |
| **AZT** | **F77S (12.5%)** | ***T215Y* (57.1%)** | **L210F, *T215Y* (6.25%)** | **None (81.8%)** | ***M41L*  (14.3%)** |
|  |  |  |  |  | ***M41L*, V75G (14.3%)** |
|  |  |  |  |  |  |
| **Visit 2007** |  |  |  |  |  |
|  | **None (100%)** |  |  |  |  |
| **HAART** |  |  |  |  |  |
|  |  |  |  |  |  |
| **Patient #8** | **PBL** | **Esophagus** | **Stomach** | **Duodenum** | **Colon** |
| **Visit 1** |  |  |  |  |  |
|  | **None (37.5%)** | ***T215Y* (50%)** | **None ( 16.7%)** | **ND** | **ND** |
| **ddI** | **K70R (12.5%)** | **None (50%)** | ***M41L*, L210W, *T215Y*  (83.3%)** |  |  |
|  | **K70R, F116L (12.5%)** |  |  |  |  |
|  | **M41I, E44K, L74V (12.5%)** |  |  |  |  |
|  | **M41I, E44K, D67N, L74V (25%)** |  |  |  |  |
|  |  |  |  |  |  |
| **Visit 2** |  |  |  |  |  |
|  | **None (100%)** | **None (100%)** | **V118I, *T215Y* (11.1%)** | **None (90.9%)** | **None (100%)** |
| **ddI** |  |  | ***M41L*, *T215Y* (11.1%)** | **T69A, F116L (9.1%)** |  |
|  |  |  | ***M41L*, L74V, *T215Y* (33.4%)** |  |  |
|  |  |  | **L74V, L210W, *T215Y (*11.1%)** |  |  |
|  |  |  | ***M41L*, V118I, L210W, *T215Y* (22.2%)** |  |  |
|  |  |  | ***M41L*, D67N, T69N, L210W, *T215Y* (11.1%)** |  |  |
|  |  |  |  |  |  |
| **Visit 3** |  |  |  |  |  |
|  | ***M41L*, D67E, L210W, *T215Y* (66.6%)** | **K65G (9.1%)** | ***T215Y* (100%)** | **ND** | **None (100%)** |
| **ddI** | ***M41L*, D67E, L210C, *T215Y* (33.4%)** | **None (0.9%)** |  |  |  |
|  |  |  |  |  |  |
| **Visit 2007** |  |  |  |  |  |
|  | ***M41L*, T215D (100%)** |  |  |  |  |
| **HAART** |  |  |  |  |  |
|  |  |  |  |  |  |
| **Patient #60** | **PBL** | **Esophagus** | **Stomach** | **Duodenum** | **Colon** |
| **Visit 1** |  |  |  |  |  |
|  | **D67N, K70R, *T215Y*, K219Q (80%)** | **None (90%)** | **None (8.3%)** | **D67N, T69N, K70R, K219Q (100%)** | **D67N, K70R, K219Q (30%)** |
| **AZT** | **D67N, K70R, K219Q (20%)** | **E44K (10%)** | ***M41L* (8.3%)** |  | **D67N, T69N, K70R, K219Q (50%)** |
|  |  |  | **K70R, *T215Y* (83.3%)** |  | **D67N, T69N, K70R, F116V, K219Q (10%)** |
|  |  |  |  |  | **E44V, D67N, T69N, K70R, K219Q (10%)** |
|  |  |  |  |  |  |
| **Visit 2** |  |  |  |  |  |
|  | **None 100%** | **D67N, T69N, K70R, K219Q (90%)** | **K70R, *T215F* (10%)** | **D67N, K70R, K219Q (50%)** | **D67N, K70R, *T215Y*, K219Q (40%)** |
| **AZT** |  | **K70R, *T215Y* (10%)** | **D67N, T69N, K70R, K219Q (10%)** | **D67N, T69N, K70R, K219Q (50%)** | **D67N, T69D, K70R, *T215Y*, K219Q (60%)** |
|  |  |  | **K70R, *T215Y* (80%)** |  |  |
|  |  |  |  |  |  |
| **Visit 3** |  |  |  |  |  |
|  | **None (90.9%)** | **D67N, T69N, K70R, K219Q (10%)** | **ND** | **None (45.5%)** | **M184T (15.4%)** |
| **ddI** | **M41T (9.1%)** | **D67G, K70R, *T215Y* (10%)** |  | **K70R, *T215Y* (18.2%)** | **D67N, T69D, K70R, *T215F*, K219Q (76.9%)** |
|  |  | **D67N, K70R, K219Q (10%)** |  | **K70R, *T215Y*, K219Q (9.1%)** | **D67N, T69D, K70R, V75I, *T215F*, K219Q (7.7%)** |
|  |  | **K70R, *T215Y* (70%)** |  | **D67N, K70R, *T215Y*, K219Q (9.1%)** |  |
|  |  |  |  | **D67N, T69D, K70R, *T215Y*, K219Q (9.1%)** |  |
|  |  |  |  | **D67N, T69N, K70R, *T215Y*, K219Q (9.1%)** |  |
|  |  |  |  |  |  |
| **Patient #19** | **PBL** | **Esophagus** | **Stomach** | **Duodenum** | **Colon** |
| **Visit 1** |  |  |  |  |  |
|  | **M41I, E44K, D67N, L74V (100%)** | **K70R (10%)** | **M41I, E44K, D67N, L74V (40%)** | **ND** | **ND** |
| **ddI** |  | **E44G (10%)** | **None (60%)** |  |  |
|  |  | **F77S (10%)** |  |  |  |
|  |  | **None (60%)** |  |  |  |
|  |  | **D67N, K70R, V118I, *T215Y,* K219Q (10%)** |  |  |  |
| **Visit 2** |  |  |  |  |  |
|  | ***M41L*, *T215Y* (100%)** | **None 77.8%** | ***T215Y* (100%)** | **None (50%)** | **F116K (14.3%)** |
| **AZT** |  | **D67G 11.1%** |  | **L74V (50%)** | **None (85.7%)** |
|  |  | **T215A 11.1%** |  |  |  |
|  |  |  |  |  |  |
| **Visit 3** |  |  |  |  |  |
|  | ***T215Y* (20%)** | ***M41L*, *T215Y* (100%)** | **None (25%)** | **None (77.8%)** | ***M41L*, L74V, *T215Y* (66.7%)** |
| **AZT** | ***M41L*, *T215Y* (80%)** |  | **K70R (50%)** | ***T215Y* (11.1%)** | **None (33.3%)** |
|  |  |  | **L210W, *T215Y* (16.7%)** | ***M41L*, D67G (11.1%)** |  |
|  |  |  | ***M41L*, L210W, *T215Y* (8.3%)** |  |  |
|  |  |  |  |  |  |
| **Visit 2007** |  |  |  |  |  |
|  | **None 9/11 (81.8%)** |  |  |  |  |
| **HAART** | ***M41L*, E44D, T215C (18.2%)** |  |  |  |  |
|  |  |  |  |  |  |
| **Patient #42** | **PBL** | **Esophagus** | **Stomach** | **Duodenum** | **Colon** |
| **Visit 1** |  |  |  |  |  |
|  | **D67N, K70R, V118I, *T215Y*, K219Q (70%)** | **K70R, *T215Y* (100%)** | **None (58.3%)** | **M184T (9.1%)** | **D67N, K70R, V118I, *T215F*, K219Q (100%)** |
| **None** | **D67N, K70R, V118I, L210F, *T215F*, K219Q (10%)** |  | **D67N, K70R, V118I, *T215Y*, K219Q (41.7%)** | **D67N, T69D, K70R, *T215F*, K219Q (45.5.%)** |  |
|  | **D67N, K70R, F116Y, V118I, *T215F*, K219Q (10%)** |  |  | **None (45.5%)** |  |
|  | **E44D, D67N, K70R, V118I, *T215F*, K219Q (10%)** |  |  |  |  |

**Additional Data Table 1: Drug resistance mutations by tissue source**

****ND - no viral sequences detected. Primary drug resistance mutations associated with high levels of drug resistance indicated in italics and red***.
